# Influence of root secretions of understory Chinese herbal medicines on the characterization of inter-root microbial communities

**DOI:** 10.3389/fpls.2025.1697347

**Published:** 2026-01-20

**Authors:** Yuan Yang, Jianqiang Li, Bo Yang, Linling Wang, Wenqi Tang, Lianling Cha

**Affiliations:** College of Soil and Water Conservation, Southwest Forestry University, Kunming, Yunnan, China

**Keywords:** under-forest *Panax notoginseng*, under-forest *Polygonatum sibiricum*, under-forest *Wasabia japonica*, root exudates, soil nutrients, soil rhizosphere microorganisms

## Abstract

The root exudates of Chinese medicinal herbs under the forest play a crucial regulatory role in the rhizosphere microbial community. This study systematically revealed the chemical characteristics of root exudates from *Panax notoginseng*, *Polygonatum sibiricum*, and *Eutrema wasabia* under the forest and their differential impact mechanisms on the rhizosphere microbial community. Research has found significant species specificity in the types and quantities of root exudates from three plants. A total of 329, 250, and 193 differential compounds were identified in *P. notoginseng*, *P. sibiricum*, and *E. wasabia*, respectively. Among them, *P. notoginseng* and *E. wasabia* share 165 compounds, both of which are mainly composed of lipids and organic acids. However, the specific types and proportions vary, reflecting their unique ecological functions and pharmacological effects. Root exudates significantly regulate the structure and diversity of microbial communities, with Proteobacteria, Acidobacteria, and Basidiomycota being dominant groups. Mantel’s test confirmed that the main components of secretions are significantly positively correlated with the abundance and diversity of microbial communities. Kyoto Encyclopedia of Genes and Genomes (KEGG) pathway enrichment analysis further showed that different plants adapt to the environment by activating differentiated metabolic pathways (22 enriched in *P. notoginseng*, 20 enriched in *P. sibiricum*, and 15 enriched in *E. wasabia*). More importantly, the microbial metabolite interaction network analysis revealed processing specificity: specific bacteria in the *P. notoginseng* forest were strongly positively correlated with lipids, and fungi with phenolic metabolites, while *P. sibiricum* and *E. wasabia* forests showed a completely different association pattern, regulating the accumulation of key metabolites such as lipids, phenols, and oligosaccharides by reshaping the network. These findings provide key evidence for understanding the microbial-driven mechanisms underlying the quality formation of medicinal plants, emphasizing the importance of optimizing root exudate management to enhance soil microbial health and promote sustainable agricultural development.

## 1 Introduction

Yunnan Province is actively promoting the development of the understory Chinese herbal medicine industry. According to the “14th Five-Year Plan of Yunnan Province”, the province makes full use of its vast forest resources to develop the cultivation of understory Chinese herbal medicines (Yunnan Provincial Forestry and Grassland Bureau, 2021). In China, it is mainly concentrated in Wenshan Zhuang and Miao Autonomous Prefecture in the southeastern part of Yunnan Province and the surrounding areas (Yang et al., 2022). Due to the serious continuous crop obstacles in herb planting and production, in recent years, Yunnan herb planting and production have begun to shift from the traditional planting areas to the surrounding areas at an accelerating rate (Huang et al., 2023). Xundian County is located in the northeast of Kunming City, Yunnan Province, which is an important expansion area of Yunnan’s herb planting and production in recent years. Based on modern spatial information technology, integrating multi-source information data, carrying out a study on the suitability evaluation of herb planting and production in Xundian County, Yunnan, and exploring the spatial distribution of different levels of suitability zones and the planning of changes, it is of great significance to speed up the construction of agricultural informatization in Xundian County and to contribute to the sustainable development of herb planting, production, and processing industries (Zhang et al., 2024).

Root secretions are generally produced through both metabolic and non-metabolic pathways (Vives-Peris et al., 2020). Primary metabolism provides energy, material, and information to enable normal plant growth and development (Canarini et al., 2022), and root secretions and secondary metabolites produced by non-metabolic pathways are an important part of chemosensory substances, which cause chemosensory autotoxicity (Li et al., 2021) and contribute to the creation of a succession of crop barriers (Zhalnina et al., 2021). As an important source of energy material for microorganisms in the inter-root soil, the type and quantity of root secretions are closely related to the type and quantity of inter-root soil microorganisms (Wang et al., 2023). It was found that the microbial community structure of the inter-root soil changed and that soil enzyme activities were closely altered due to the autotoxic substances produced by the continuously accumulating root secretions and residue degradation after plant succession (Chen et al., 2022). The decrease in soil enzyme activity will hinder the nutrient uptake of herbs and intensify the accumulation of self-toxic substances in the soil, inducing the production of continuous cropping disorder in herbs. It was found that there is an interaction between the inter-root environment of plants and root secretions (Zhang et al., 2020). On the one hand, changes in the inter-root environment in which the plants are located will affect the type and amount of inter-root secretions, and on the other hand, inter-root secretions will also cause changes in the inter-root environment. Therefore, root secretion as a medium of interaction between plants, soil and inter-root microorganisms (Sasse et al., 2022), the secretion process in the inter-root soil will cause changes in soil physicochemical properties and microbial community structure (Liu et al., 2021); changes in microbial community will indirectly contribute to changes in soil enzyme activities and ultimately changes in the soil microbiological environment (Qin et al., 2024b), where chemosensory self-toxicity will produce chemosensory self-toxicity (Burns et al., 2021; Wu et al., 2023b), inhibiting the normal growth of plants; the ecological effect of these factors together in the inter-root environment is an important condition for the generation of continuous crop disorder in herbs (Wu et al., 2023a).

The inter-root soil microbial community plays an important role in plant growth and development, stress resistance, and crop yield as a regulator of soil health, crop productivity, and sustainability of cultivated plants (Trivedi et al., 2022). Alterations in the structure and function of the inter-root soil microbial community of medicinal herbs are closely related to the occurrence of the soil-borne disease root rot and succession barriers (Liu et al., 2022), and the mechanism of the role of root secretions in the characterization of soil microbial communities is of great importance for forest herbs as a new cultivation mode (Qin et al., 2024a). Research in this field can help to reveal the soil microbial ecological changes during the growth of understory herbs and provide a scientific basis for the rational utilization of understory herb resources and the improvement of soil quality and ecological benefits (Kaur et al., 2020). In addition, the results of the study have a wide range of application value in the fields of herbal medicine cultivation, ecological environmental protection, and soil health management (Philippot et al., 2022).

## 2 Materials and methods

### 2.1 Overview of the test site

The experimental site was located in Yunnan Agricultural University Experimental Demonstration Base for Understory Chinese Materia Medica Planting, Xundian County, Kunming City, Yunnan Province, China. The study area had a subtropical monsoon climate with obvious rainy and dry seasons. The altitude of the study area was 2,210 m, and the soil was red loam acidic soil. The average annual rainfall was 1,254 mm, and it was mostly concentrated in May–December, with sufficient sunshine and a large temperature difference. The average annual temperature was 14.68°C, the highest temperature was 37.06°C, and the lowest temperature was 2.53°C. The main trees in the test area were *Pinus yunnanensis*, with a closure degree of 0.7, an average height of 17.5 m, and an average diameter at breast height (Dbh) of 27 cm. The understory shrubs were *Hypericum monogynum*, *Schima wallichii*, *Vaccinium bracteatum*, and herbaceous plants, mainly including fragrant *Isodon amethystoides*, *Ageratina adenophora*, and *Oplismenus compositus*.

### 2.2 Experimental design and sampling methods

In this study, natural forest land (RCK) was used as the control group; forest land planted with *Panax notoginseng*, *Polygonatum sibiricum*, and *Eutrema wasabia* in the understory was selected as the study object; their soil properties were analyzed separately. Sampling was carried out in the rainy season of 2023, and the collection sites included natural woodland (RCK), forest plantation of *P. notoginseng* (RPN), forest plantation of *P. sibiricum* (RPS), and forest plantation of *E. wasabia* (REW), among which the first three groups were selected as the control group, of which the first three groups were official samples and the last three groups were backup samples. All samples were collected from the 0–20-cm soil layer, and a total of 15 soil samples were collected. The sampling tools were autoclaved before sampling, and the soil samples were put into sterile sealed bags immediately after collection, stored at −20°C, and brought back to the laboratory quickly. Each soil sample was divided into two parts: one part was used for the characterization of the soil microbial community, and the other part was air-dried, ground, and sieved for the determination of soil physicochemical properties.

### 2.3 Determination of physical and chemical properties of soil

The determination of key soil nutrient properties was conducted using a series of established analytical methods. Total nitrogen was quantified via the Kjeldahl method, which involved an initial digestion of the sample with concentrated sulfuric acid, followed by automated analysis. Total phosphorus was determined by first digesting the samples with a perchloric acid–sulfuric acid mixture, with the resulting solution then analyzed using a continuous flow analyzer. For the measurement of total potassium, a sodium hydroxide alkali fusion-flame photometry method was employed; this procedure consisted of high-temperature fusion of the sample with NaOH, followed by the quantification of potassium concentration using a flame photometer to measure the element’s characteristic spectral radiation. The content of available phosphorus was ascertained by leaching the soil with a sodium bicarbonate solution, after which the extract was also measured using a continuous flow analyzer. Finally, available potassium was determined by leaching the samples with an ammonium acetate solution, and the concentration in the extract was subsequently quantified using flame photometry.

### 2.4 Measurement of root exudates

One hundred milligrams of the sample was accurately weighed and placed in a 2-mL centrifuge tube. One 6-mm grinding bead was added. Eight hundred microliters of a methanol–water (4:1, v/v) solution containing four internal standards (such as l-2-chlorophenylalanine 0.02 mg/mL) was extracted. The sample was first frozen and ground for 6 minutes (−10°C, 50 Hz) and then subjected to low-temperature ultrasound for 30 minutes (5°C, 40 kHz). After allowing the extract to stand at −20°C for 30 minutes, it was centrifuged at 4°C and 13,000 *g* for 15 minutes, and the supernatant was transferred to an injection bottle containing an inner liner for testing.

Chromatographic separation: 3 μL of the sample was separated on a BEH C18 column (100 mm × 2.1 mm i.d., 1.7 μm) at a flow rate of 0.40 mL/min and column temperature of 40 °C. Mobile phase A was a 0.1% formic acid aqueous solution (containing 2% acetonitrile), and phase B was 0.1% formic acid acetonitrile. Gradient elution program: at 0–0.5 min, phase B was 2%; at 0.5–7.5 minutes, phase B increased to 35%; at 7.5–13 minutes, phase B increased to 95%; at 13–14.4 minutes, phase B remained at 95%; at 14.4–14.5 minutes, phase B decreased to 2%; at 14.5–16 minutes, phase B was at 2% equilibrium. Mass spectrometry collection: positive and negative ion switching scanning, m/z 70–1,050. Ion source parameters: positive and negative voltage 3,500 V/−3,000 V, sheath gas 50 psi, auxiliary gas 13 psi, and temperature 450°C. The collision energy adopted a 20–40–60 V step mode, with MS1/MS2 resolution set to 70,000/17,500.

### 2.5 Determination of soil microbial communities

For total genomic DNA extraction from microbial communities, DNA extraction was first performed according to the instructions of the E.Z.N.A.^®^ soil DNA kit (Omega Bio-tek, Norcross, GA, USA). After extraction, the quality of the DNA was checked using 1% agarose gel electrophoresis. During electrophoresis, DNA samples should form a clear single band, indicating that the DNA is not degraded and that there are no impurities in the sample. The integrity of the DNA was confirmed by observation at 260 nm using a UV transilluminator. After electrophoresis, the concentration and purity of the DNA were determined using a NanoDrop2000 (Thermo Fisher Scientific, Shanghai). The ideal A260/A280 ratio should be between 1.8 and 2.0, which indicates good DNA purity. Using the extracted DNA described above as a template, the upstream primer 338F (5′-ACTCCTACGGGGAGGCAGCAG-3′) carrying the barcode sequence and the downstream primer 806R (5′-GGACTACHVGGGTWTCTAAT-3′) were used for the PCR amplification of the V3–V4 variable region of the 16S rRNA gene. The PCR reaction mixture including 4 µL 5 × Fast Pfu buffer, 2 µL 2.5 mM dNTPs, 0.8 µL each primer (5 µM), 0.4 µL Fast Pfu polymerase, 10 ng of template DNA, and ddH2O to a final volume of 20 µL. The amplification procedure was as follows: 95°C pre-denaturation for 3 min, 27 cycles (95 °C denaturation for 30 s, 55°C annealing for 30 s, and 72°C extension for 30 s), and then 72°C stabilization and extension for 10 min. Finally, it was stored at 4°C (PCR instrument: ABI GeneAmp^®^ 9700). The PCR products were recovered on a 2% agarose gel, purified using the PCR Clean-Up Kit (China), and quantified using Qubit 4.0 (Thermo Fisher Scientific, USA).

The purified PCR products were used for library construction using the NEXTFLEX Rapid DNA-Seq Kit: 1) splice linkage, 2) removal of splice self-associated fragments by magnetic bead screening, 3) enrichment of library templates by PCR amplification, and 4) recovery of PCR products by magnetic beads to obtain the final library. Sequencing was performed using the Illumina PE300/PE250 platform (Shanghai Meiji Biomedical Technology Co., Ltd., Pudong New Area, Shanghai).

### 2.6 Data processing and analysis

Excel 2016 was used for data organization, and SPSS Statistics 24.0 one-way analysis of variance (ANOVA; Lysergic Acid Diethylamide (LSD), p < 0.05) was used to analyze the differences between different treatments. GraphPad Prism 9.5 was used for plotting.

## 3 Result

### 3.1 Analysis of root secretion profiles in understory medicinal plants

#### 3.1.1 Dissimilarity and principal component analysis of root secretions

**Figure 1:**
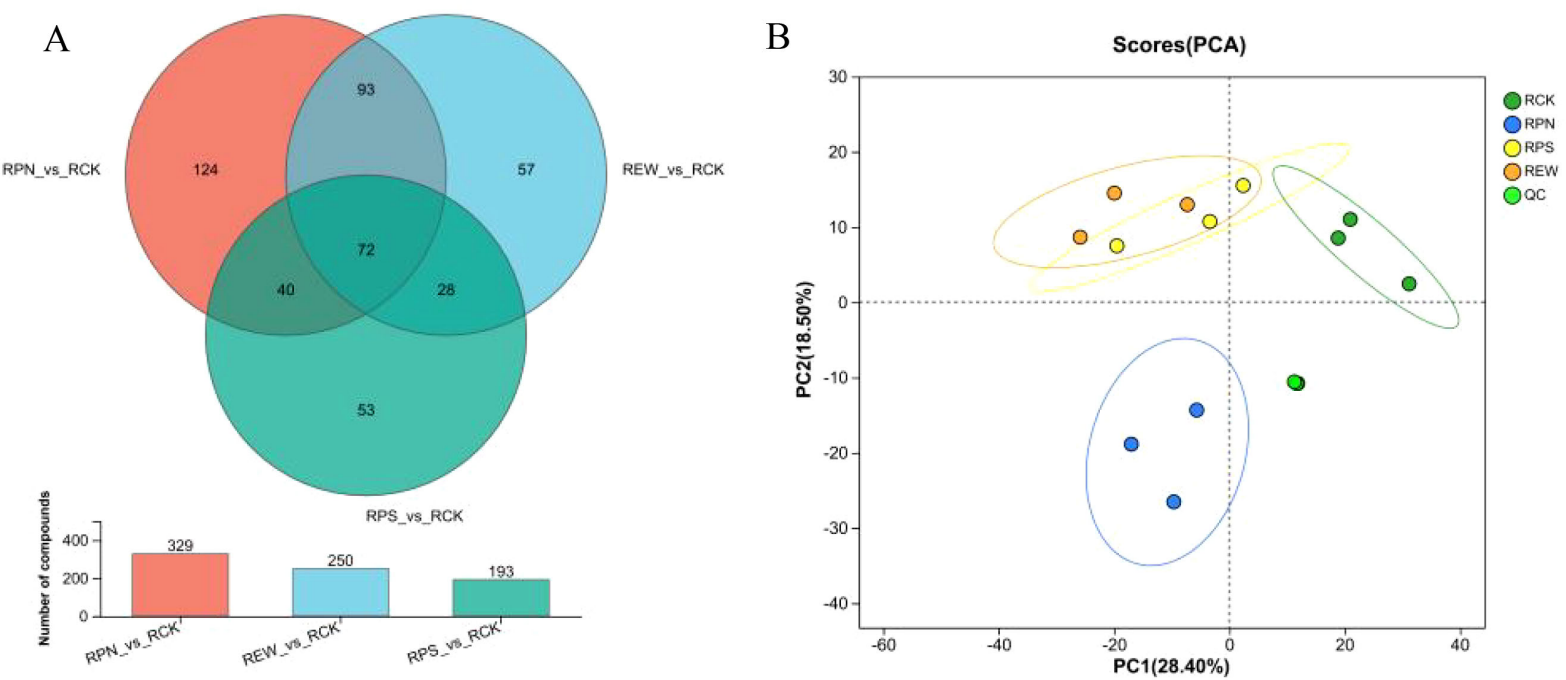
Comparative analysis of root secretions from understory medicinal plants. **(A)** Venn diagram of root secretion components. **(B)** Principal component analysis (PCA) of root secretions.

There were 329, 250, and 193 significantly different secretions for understory *P. notoginseng*, *P. sibiricum*, and *E. wasabia*, respectively, compared to the unplanted control (**Figure 1A**). Between understory *P. notoginseng* and *E. wasabia*, there were 165 significantly different secretions together, including 1-(2,3-dihydro-1*H*-inden-2-ylamino)-3-(3,4-dimethylphenoxy)propan-2-ol, 4-anisic acid, D1-3-hydroxykynurenine, *N*-palmitoyl taurine, and *N*1-(2-methyl-4-nitrophenyl)acetamide. Between *P. notoginseng* and *P. sibiricum*, there were 112 common differential secretions, consisting mainly of 4-anisic acid, 4-hydroxycoumarin, kynurenine *O*-hexoside, L-macrogalline N-acetyl-L-histidine, N-formylmorpholine, and *N*,6-diphenylthieno[2,3-*d*]pyrimidin-4-amine. Between the forest *E. wasabia* and *P. sibiricum*, there were 100 common differential secretions, including 15-deoxy-Δ12,14-prostaglandin D2, 3-[4-(*tert*-butyl)anilino]-2-(3-thienylcarbonyl)acrylonitrile, 4-hydroxytamoxifen, 4-anisic acid, and 5,7-dihydroxy-3,8-dimethoxy-2-phenyl-4*H*-benzopyran-4-one. In addition, a total of 72 identical differential secretions, mainly 4-anisic acid, were found in all three compared to the unplanted control.

In order to accurately analyze the variability of root secretions of different understory herbs, orthogonal partial least squares discriminant analysis (OPLS-DA) was used (**Figure 1B**). The analysis results indicated that RPN, RPS, REW, and RCK were significantly different in root secretions. Therefore, it can be concluded that the root secretion of *P. notoginseng*, *P. sibiricum*, and *E. wasabia* in the forest was significantly different compared to the unplanted group.

#### 3.1.2 Classification of HMDB compounds in root secretions of understory medicinal plants

Root secretions of *P. notoginseng*, *P. sibiricum*, and *E. wasabia* were identified separately using Liquid Chromatography-Mass Spectrometry (LC-MS).

**Figure 2:**
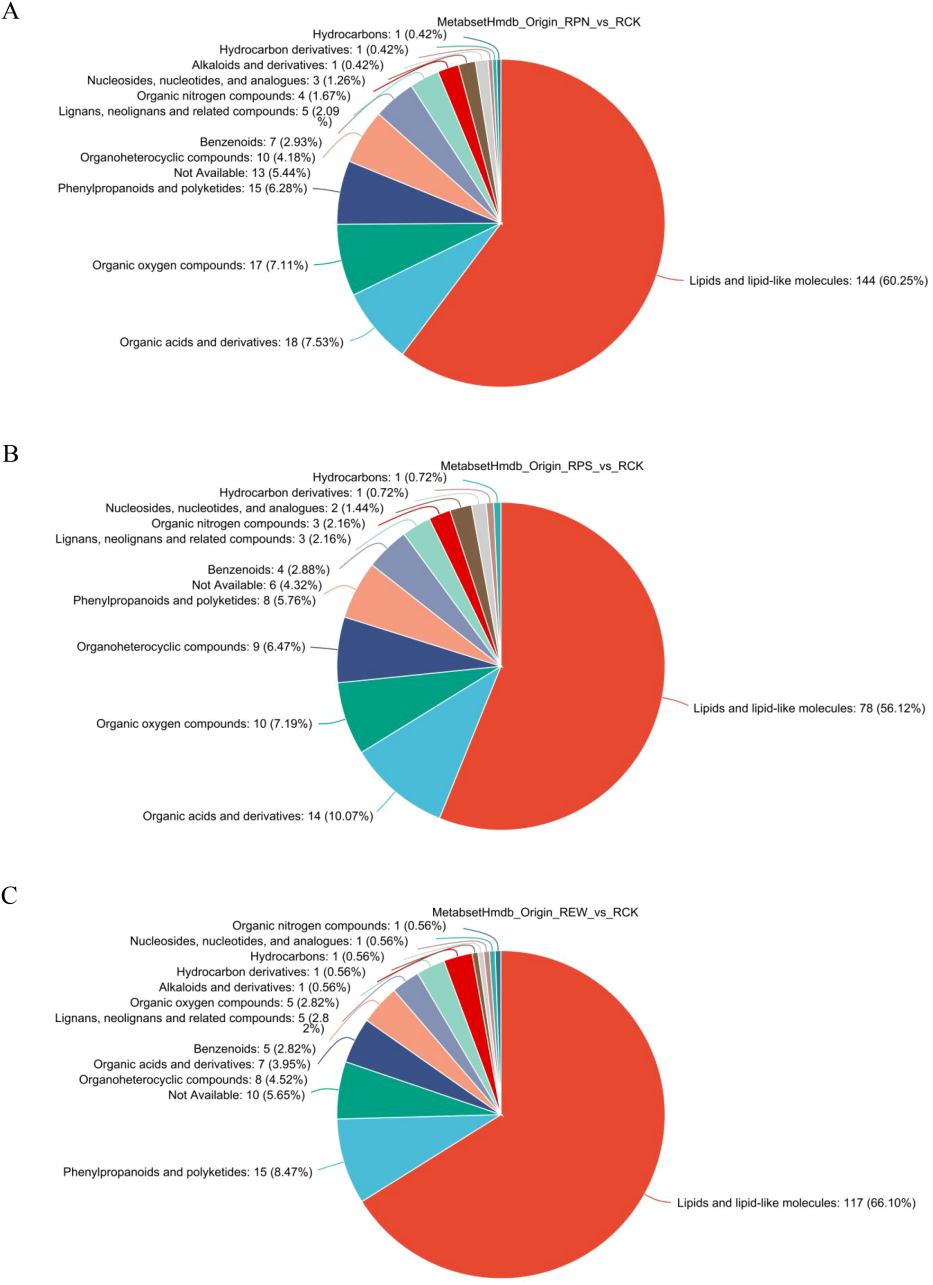
Classification of Human Metabolome Database (HMDB) compounds in root secretions of understory medicinal plants. **(A)** Human Metabolome Database (HMDB) compound classification in *Panax notoginseng* root secretions. **(B)** HMDB compound classification in *Polygonatum sibiricum* root secretions. **(C)** HMDB compound classification in *Eutrema wasabia* root secretions.

A total of 238 substances were detected in *P. notoginseng* root secretions. These substances belonged to 13 categories, including lipids and lipid-like molecules; organic acids and their derivatives; organic oxides; phenylpropanoids and polyketides; others; organic heterocyclic compounds; benzene compounds; lignans, neolignans, and related compounds; organic nitrogen compounds; nucleosides, nucleotides, and their analogs; alkaloids and their derivatives; hydrocarbon derivatives; and aliphatic hydrocarbons (**Figure 2A**). Lipids and lipid-like molecules, organic acids and their derivatives, organic oxides, phenylpropanoids and polyketides, and others had relatively high relative contents of 60.25%, 7.53%, 7.11%, 6.28%, and 5.44%, respectively. In contrast, the relative contents of organic heterocyclic compounds; benzene compounds; lignans, neolignans, and related compounds; organic nitrogen compounds; nucleosides, nucleotides, and their analogs; alkaloids and their derivatives; hydrocarbon derivatives; and aliphatic hydrocarbons were relatively low, ranging from 0.42% to 4.18%.

In *P. sibiricum*, 139 substances were detected. They mainly consisted of 12 types of substances, namely, lipids and lipid-like molecules; organic acids and their derivatives; organic oxygen compounds; organic heterocyclic compounds; phenylpropanoids and polyketides (an unclassified category); benzene compounds; lignans, neolignans, and related compounds; organic nitrogen compounds; nucleosides, nucleotides, and their analogs; derivatives of hydrogen-containing compounds; and hydrogenated carbon compounds (**Figure 2B**). Lipids and lipid-like molecules accounted for the largest proportion (56.12%), followed closely by organic acids and their derivatives (10.07%), organic heterocyclic compounds (7.19%), phenylpropanoids and polyketides (6.47%), and other substances (5.76%). The proportions of hydrogenated carbon compounds; derivatives of hydrogen-containing compounds; nucleosides, nucleotides, and their analogs; organic nitrogen compounds; lignans, neolignans, and related compounds; and benzene compounds were lower, ranging from 0.72% to 4.32%.

A total of 162 substances were detected in the root secretions of *E. wasabia*, belonging to 11 categories such as lipids and lipid-like molecules; phenylpropanoids and polyketides; organic heterocyclic compounds; organic acids and their derivatives; benzene compounds; organic oxygen compounds; alkaloids and their derivatives; hydrocarbon derivatives; nucleosides, nucleotides, and their analogs; hydrocarbons; and organic nitrogen compounds (**Figure 2C**). Lipids and lipid-like molecules had the highest relative content of 66.10%, followed by phenylpropanoids and polyketides with 8.47%. Organic heterocyclic compounds, organic acids and their derivatives, benzene compounds, and organic oxygen compounds had relative contents of 4.52%, 3.95%, 2.82%, and 2.82%, respectively. The relative contents of alkaloids and their derivatives; hydrocarbon derivatives; nucleosides, nucleotides, and their analogs; hydrocarbons; and organic nitrogen compounds were low, all approximately 0.56%.

The results of the LC-MS identification of the root secretions of *P. notoginseng*, *P. sibiricum*, and *E. wasabia* showed the commonalities and differences in the chemical constituents of these plants. For commonality, lipids and lipid-like molecules occupied a large proportion in all three plants, namely, *P. notoginseng* (60.25%), *P. sibiricum* (56.12%), and *E. wasabia* (66.10%), which suggests that lipid-like compounds play an important role in their metabolic processes. Another commonality was the high relative content of organic acids and their derivatives in all three, which was 7.53% in *P. notoginseng*, 10.07% in *P. sibiricum*, and 3.95% in *E. wasabia*. In addition, the presence of organic oxygenates (7.11%, 7.19%, and 2.82%, respectively) and phenylpropanoids and polyketides (6.28%, 5.76%, and 8.47%, respectively) in all three showed the potential roles that these substances may play in physiological functions, such as those in antioxidants. Concerning variability, the three species differed significantly in terms of the types and proportions of substances. With 238 substances detected, *P. notoginseng* had the largest variety of chemical constituents, compared with 139 and 162 substances for *P. sibiricum* and *E. wasabia*, respectively. Especially in the distribution of components, the proportion of organic heterocyclic compounds in *P. notoginseng* (0.42%) was much lower than that in *P. sibiricum* (7.19%) and *E. wasabia* (4.52%). In addition, the three plants differed in the proportions of phenylpropanoid compounds, which were 2.88% in *P. notoginseng*, 2.82% in *E. wasabia*, and 1.44% in *P. sibiricum*. The proportion of phenylpropanoids and polyketides was significantly higher in *E. wasabia* (8.47%) than in *P. notoginseng* (6.28%) and *P. sibiricum* Odorati (5.76%). These differences suggest that the three plants may have different physiological functions and adaptive strategies in the distribution of chemical constituents and metabolic pathways.

In summary, *P. notoginseng*, *P. sibiricum*, and *E. wasabia* have complex compositional composition, and they have commonality in components such as lipids and organic acids, but there are significant differences among the three species in terms of the types and proportions of the components, especially in the contents of organic heterocyclic compounds, benzene compounds, and phenylpropanoid and polyketone compounds. These differences may be closely related to their ecological functions, pharmacological effects, and metabolic characteristics.

#### 3.1.3 KEGG pathway enrichment analysis of root secretions in understory medicinal plants

**Figure 3:**
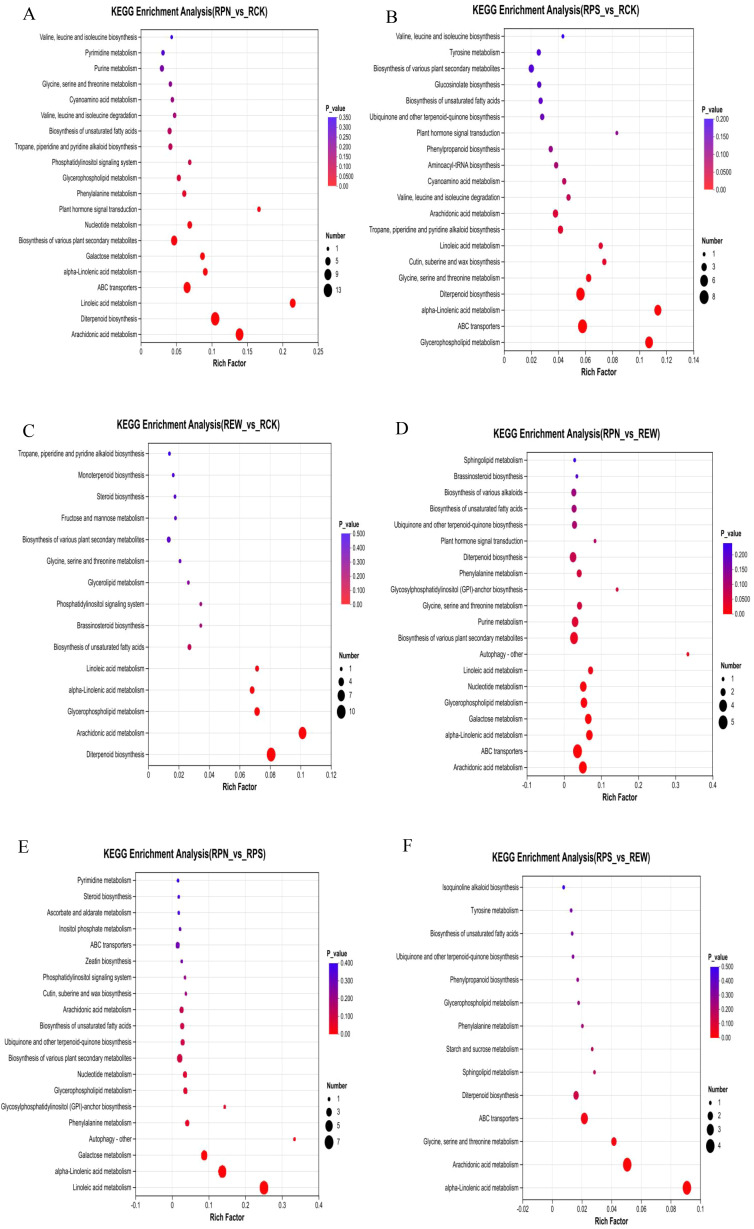
KEGG pathway enrichment analysis of root secretions from understory medicinal plants. **(A)** Enriched pathways in *Panax notoginseng* compared to natural woodland. **(B)** Enriched pathways in *Polygonatum sibiricum* compared to natural woodland. **(C)** Enriched pathways in *Eutrema wasabia* compared to natural woodland. **(D)** Enriched pathways in *P. notoginseng* compared to *E*. *wasabia*. **(E)** Enriched pathways in *P. notoginseng* compared to *P. sibiricum*. **(F)** Enriched pathways in *P. sibiricum* compared to *E*. *wasabia*.

A total of 22 metabolic pathways were enriched in the understory *P. notoginseng* plantation (RPN) compared to the natural woodland (RCK) with 14 significant metabolic pathways (p < 0.05), namely, pyrimidine metabolism; purine metabolism; aminoacyl-tRNA biosynthesis; glycine, serine, and threonine metabolism; cyanoamino acid metabolism; valine, leucine, and isoleucine degradation; unsaturated fatty acid biosynthesis; tropane, piperidine, and pyridine alkaloid biosynthesis; phosphatidylinositol signaling system; glycerophospholipid metabolism; phenylalanine metabolism; phytohormone signaling; nucleotide metabolism; and biosynthesis of various plant secondary metabolites. Among the significantly enriched pathways, pyrimidine metabolism and purine metabolism were the most highly enriched, while phytohormone signaling and phenylalanine metabolism were also represented by a representative number of genes (**Figure 3A**).

A total of 20 metabolic pathways were enriched in the forested *P. sibiricum* plantation (RPS) compared to the natural woodland (RCK). Among them, the 10 significant metabolic pathways (p < 0.05) were valine, leucine, and isoleucine biosynthesis; tyrosine metabolism; biosynthesis of various plant secondary metabolites; glucoside biosynthesis; unsaturated fatty acid biosynthesis; ubiquinone and other terpene quinone biosynthesis; phytohormone signaling; phenylpropanoid biosynthesis; aminoacyl-tRNA biosynthesis; and cyanoamino acid metabolism. Among them, valine, leucine, and isoleucine biosynthesis and tyrosine metabolism were highly enriched, while the number of genes for phenylalanine class biosynthesis and phytohormone signaling showed high representation. This indicates that significant metabolic pathways in RPS are mainly concentrated in functional pathways related to amino acid metabolism, secondary metabolites, and fatty acid synthesis (**Figure 3B**).

A total of 15 metabolic pathways were enriched in the forested *E. wasabia* plantation woodland (REW) compared to the natural woodland (RCK). Among them, there were five significant metabolic pathways (p < 0.05): diterpene biosynthesis, arachidonic acid metabolism, glycerophospholipid metabolism, α-linolenic acid metabolism, and linoleic acid metabolism. Among them, valine and leucine were highly enriched, and the number of genes showed high representation. This indicates that the significant metabolic pathways in REW mainly focus on the functional pathways related to valine and leucine synthesis (**Figure 3C**).

A total of 20 metabolic pathways were enriched in the forest plantation of *Panax pseudoginseng* (RPN) compared to the forest plantation of *E. wasabia* (REW). Among them, the 14 significant metabolic pathways (p < 0.05) were phenylalanine metabolism; glycosylphosphatidylinositol (GPI)-anchored biosynthesis; glycine, serine, and threonine metabolism; purine metabolism; biosynthesis of various plant secondary metabolites; autophagy-others; linoleic acid metabolism; nucleotide metabolism; glycerophospholipid metabolism; galactose metabolism; α-linolenic acid metabolism; ABC transport proteins; arachidonate metabolism; and arachidonic acid metabolism. Among them, autophagy-others was highly enriched, while the number of genes for ABC transporter proteins, arachidonic acid metabolism, and biosynthesis of various plant secondary metabolites showed a high representation (**Figure 3D**).

A total of 20 pathways were enriched in the forested *P. pseudoginseng* plantation (RPN) compared to the forested *P. sibiricum* plantation (RPS). Among them, there were eight significant metabolic pathways (p < 0.05): nucleotide metabolism, glycerophospholipid metabolism, glycosylphosphatidylinositol (GPI)-anchored biosynthesis, phenylalanine metabolism, autophagy-others, galactose metabolism, α-linolenic acid metabolism, and linoleic acid metabolism. Linoleic acid metabolism and autophagy-others were highly enriched, while α-linolenic acid metabolism and linoleic acid metabolism showed a high representation in the number of genes (**Figure 3E**).

A total of 14 pathways were enriched in the understory *E. wasabia* plantation woodland (REW) compared to the understory *P. sibiricum* plantation woodland (RPS). Among them, there were four significant metabolic pathways (p < 0.05): pyrimidine metabolism, nucleotide metabolism, linoleic acid metabolism, and glycolysis and gluconeogenesis. Among them, linoleic acid metabolism and glycolysis and gluconeogenesis were highly enriched, and the number of genes showed high representation (**Figure 3F**).

The enrichment characteristics of metabolic pathways varied among different understory plants. *P. pseudoginseng* (RPN) plantation showed high metabolic diversity, mainly enriching pathways related to nucleotide metabolism, amino acid metabolism (e.g., pyrimidine and purine metabolism) and lipid metabolism (e.g., glycerophospholipid metabolism and linoleic acid metabolism); *P. sibiricum* (RPS) plantation was concentrated in amino acid metabolism, fatty acid synthesis, and secondary metabolite synthesis, especially in amino acid synthesis and phytohormone signal transduction. The plantation of *E. wasabia* (REW) was mainly enriched in pathways related to diterpene biosynthesis, fatty acid metabolism (e.g., linoleic acid metabolism), and glycerophospholipid metabolism, indicating a strong dependence on these metabolic pathways.

### 3.2 Community characteristics of inter-root microorganisms in forested Chinese herbal soils

#### 3.2.1 Composition of soil microbial communities in understory medicinal plants

**Figure 4:**
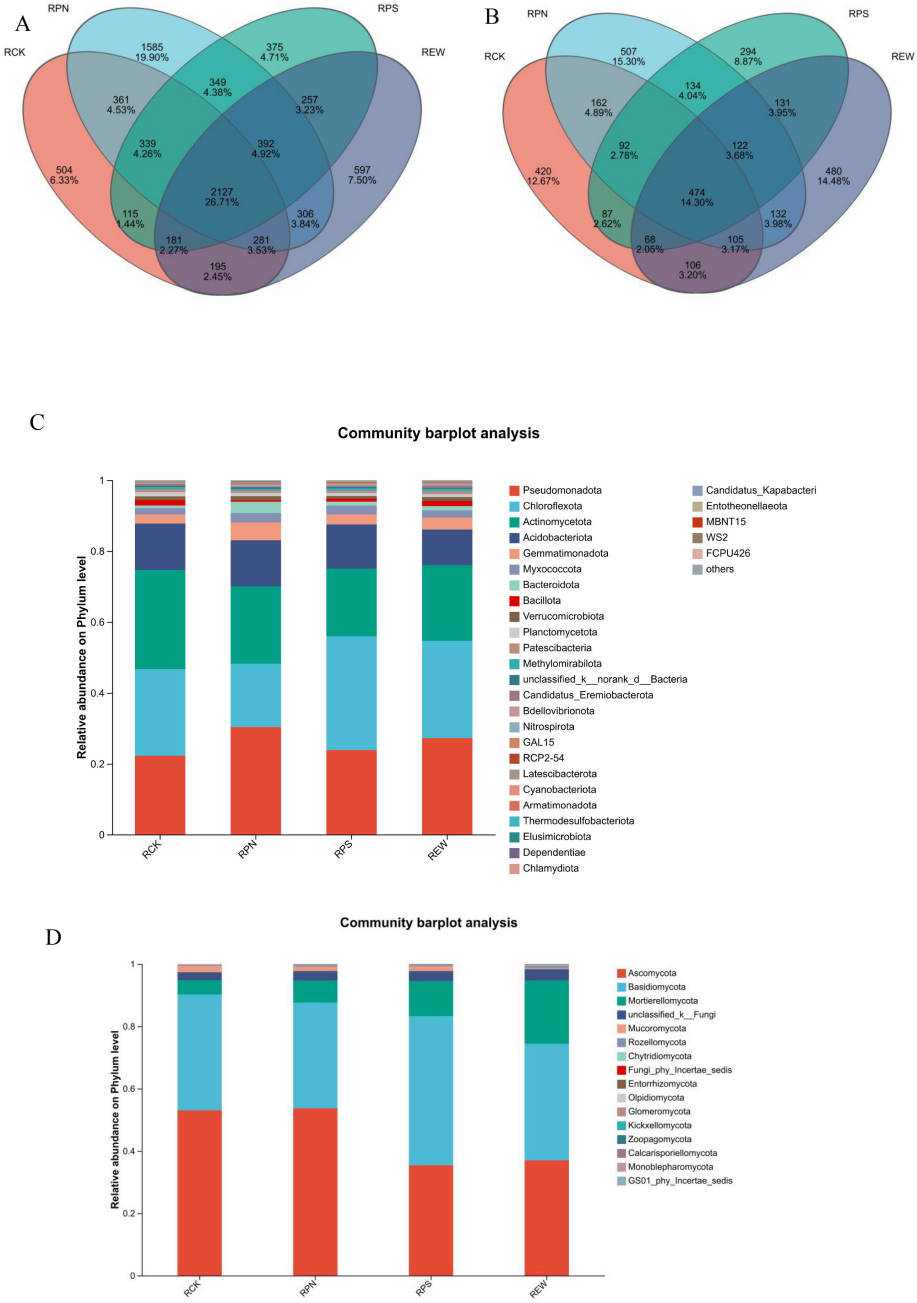
Analysis of soil microbial community composition and relative abundance in understory medicinal plants. **(A)** Venn diagram of bacterial community composition. **(B)** Venn diagram of fungal community composition. **(C)** Relative abundance of bacterial phyla. **(D)** Relative abundance of fungal phyla.

In this study, based on the distribution of operational taxonomic units (OTUs) of bacteria and fungi as well as the analysis of taxonomic abundance, the ecological characteristics of four groups of soil microbiota (namely, RPN, RCK, RPS, and REW) and their significant differences in different environments and seasons were revealed. The results of the Venn diagrams showed that the four groups of shared OTUs of the bacterial community accounted for up to 29.03%, indicating that there were obvious shared dominant bacterial groups among different soil types, with RCK and REW having the highest number of group-specific OTUs (5.81% and 6.32%, respectively). In contrast, the share of shared OTUs in the fungal community was only 15.91%, and RPN and REW had a higher proportion of group-specific OTUs (14.73% and 12.80%, respectively), indicating that the distribution of the fungal communities in different soils was more heterogeneous (**Figures 4A, B**). At the phylum classification level, the bacterial groups Proteobacteria, Acidobacteriota, Chloroflexi, and Actinobacteriota are the dominant phyla. In the cultivation environment (REW group), the relative abundance of Proteobacteria and Acidobacteriota is significantly higher than that in the natural environment (RCK group), while the relative abundance of Chloroflexi and Actinobacteriota is significantly elevated in the cultivation environment (P < 0.05). Basidiomycota, Ascomycota, and Mortierellomycota were the main dominant phyla in the fungal group, in which the relative abundance of Ascomycota was significantly higher in the planted environment than that in the natural environment (p < 0.05). However, the abundance of Basidiomycota was significantly more dominant in the natural environment (**Figures 4C, D**). Seasonal factors also had an influence on community composition; for example, the abundance of the Ascomycetes phylum showed significant variability between the dry and rainy seasons. The results of this study highlight that the significant differences in the structure and abundance of bacterial and fungal communities in natural control and planted woodlands may be strongly driven by planting activities and changes in environmental conditions, providing an important scientific basis for revealing the ecological response mechanisms of microbial communities.

#### 3.2.2 Microbial diversity in soils of understory medicinal plants

**Figure 5:**
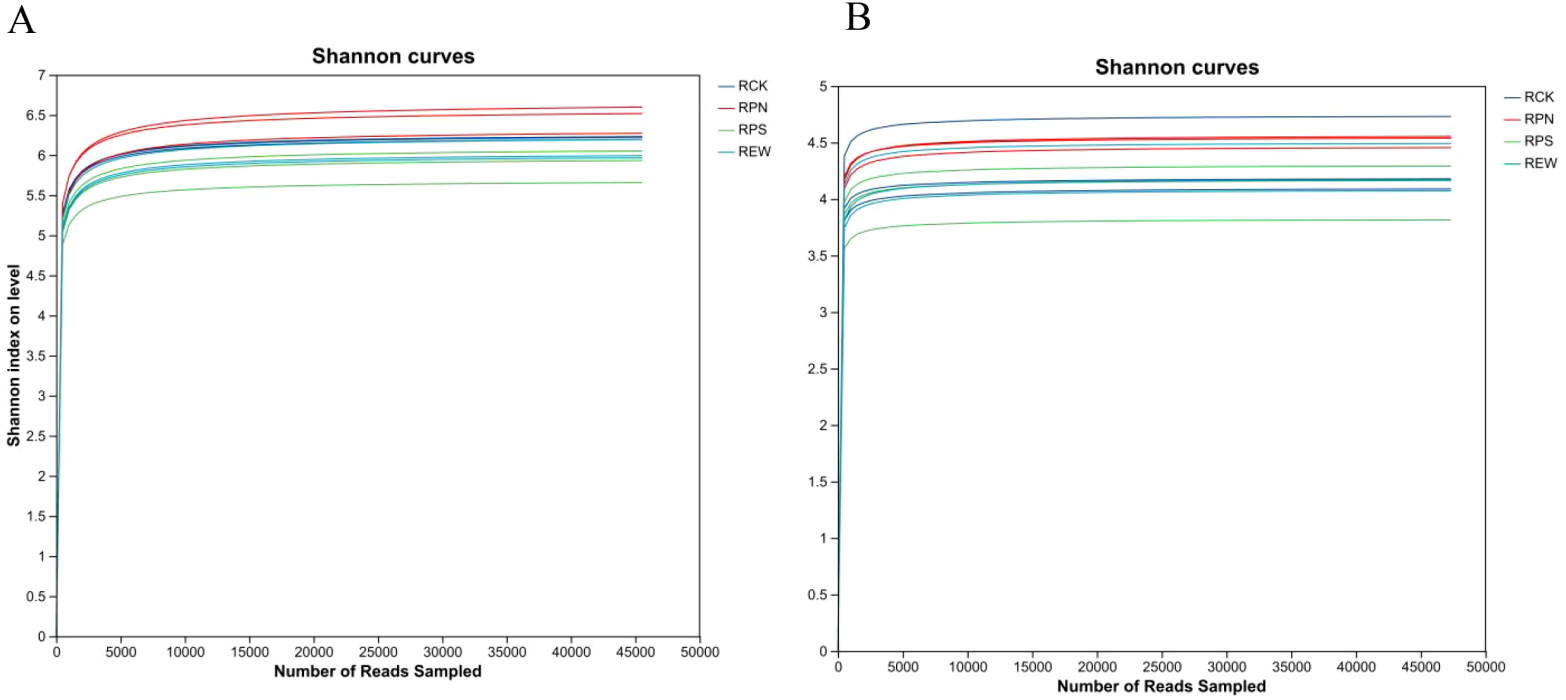
Microbial diversity rarefaction curves for understory medicinal plants. **(A)** Bacterial diversity rarefaction curves. **(B)** Fungal diversity rarefaction curves.

**Figure 5** shows the Shannon diversity dilution curve of the microbial community of medicinal plants under the forest, revealing the microbial community structure characteristics of different treatment groups (RCK, RPN, RPS, and REW) through the relationship between sequencing depth and diversity index of bacteria (**Figure 5A**) and fungi (**Figure 5B**). The bacterial diversity curve (**Figure 5A**) shows that the Shannon index of the RPN group was the highest (approximately 6.6), significantly higher than that of the control group RCK (approximately 6.2) and REW group (approximately 6.1), while the RPS group had the lowest index (approximately 5.8), indicating that its bacterial community diversity was inhibited. In the fungal diversity curve (**Figure 5B**), the RPN group had the highest index (approximately 4.6), the RPS group had the lowest index (approximately 3.8), and the fungal diversity in each group was lower than that of bacteria. All curves tended to flatten out, confirming that the sequencing depth of 50,000 reads was sufficient to reflect community structure. RPN treatment significantly enhanced microbial diversity, while RPS treatment inhibited diversity, suggesting that different treatments affected microbial community composition by regulating environmental factors or plant secondary metabolites.

**Figure 6:**
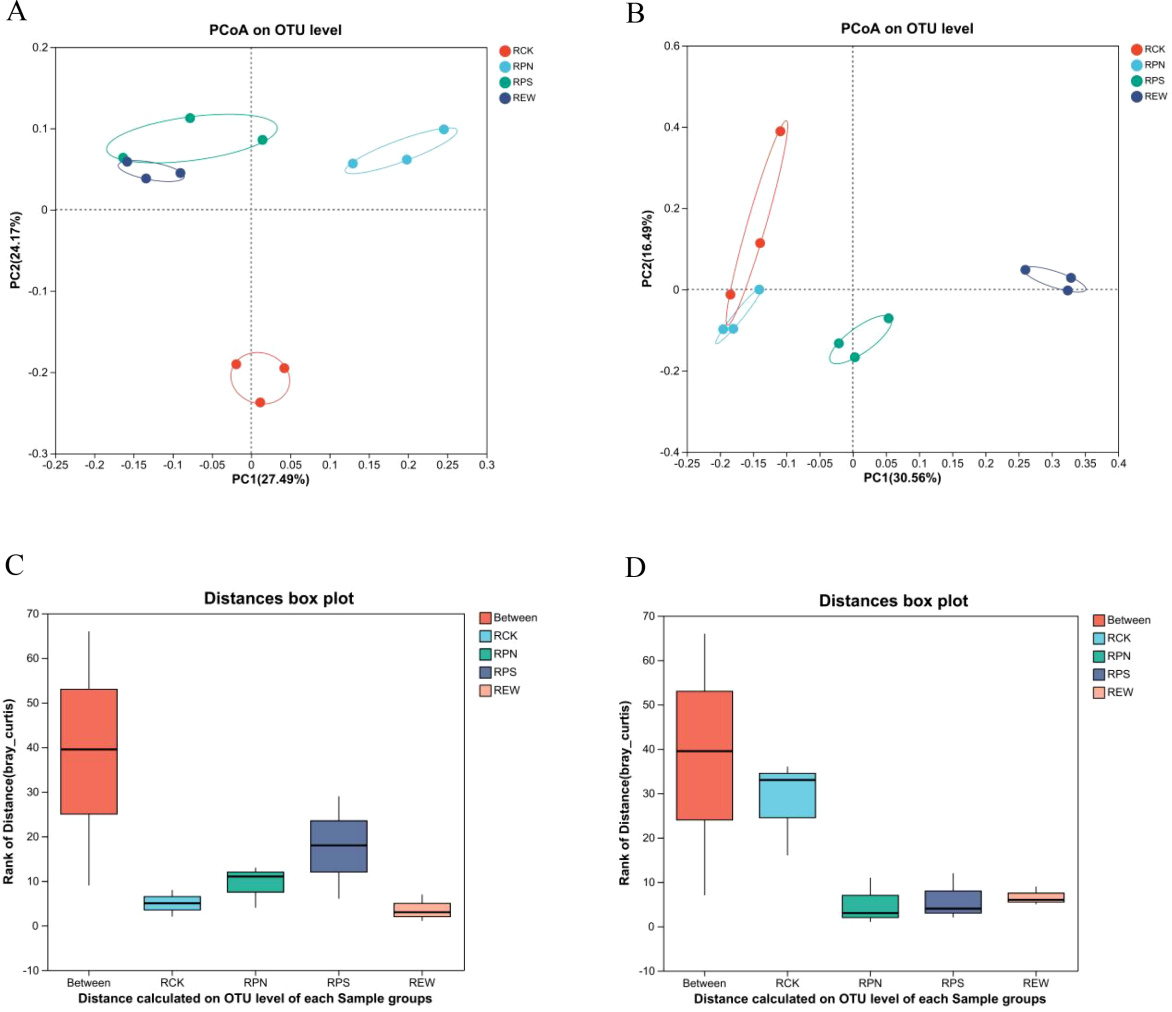
PCoA and ANOSIM analysis of soil microbial communities at the OTU level. **(A)** PCoA results for bacterial communities. **(B)** PCoA results for fungal communities. **(C)** ANOSIM results for bacterial communities. **(D)** ANOSIM results for fungal communities. PCoA, principal coordinate analysis; OUT, operational taxonomic unit.

Principal coordinate analysis (PCoA) based on OTU levels using the Bray–Curtis algorithm to assess the beta diversity of soil microbial communities showed that bacterial (**Figure 6A**) and fungal (**Figure 6B**) communities were significantly differentiated in community structure between soil treatment groups. In PCoA, PC1 and PC2 principal coordinate axes of bacteria explained 27.49% and 17.17% of the variance, respectively, and the PC1 and PC2 principal coordinate axes for fungi explained 30.56% and 16.49% of the variance, respectively. In the bacterial community, the RPS and RPN groups had a closer distribution of community structure, while the RCK and REW groups had significant differences in community structure, and both showed independent clustering. Among the fungal communities, the community structure of the RPS group was more dispersed, while the RCK group was more aggregated, indicating that the soil treatments significantly affected the structural composition of the fungal communities.

Further intra- and inter-group differences were assessed by distance box line plots, which showed that inter-group distances were significantly higher than intra-group distances for both bacterial (**Figure 6C**) and fungal (**Figure 6D**) communities, and that the RPS group showed greater fluctuations in the diversity of microbial community structure. Analysis of Similarities (ANOSIM) analysis showed that the inter-group R-value for the bacterial community was 0.312 (p = 0.001). The intergroup R-value was 0.428 (p = 0.001) for the fungal community, indicating that the effect of different treatments on microbial community structure was statistically significant. These results suggest that different treatment conditions of soil have important effects on the beta diversity of both bacterial and fungal communities, providing new evidence for understanding the response of microbial communities to environmental changes.

#### 3.2.3 Correlation between soil physicochemical properties and microbial communities in understory medicinal plants

**Figure 7:**
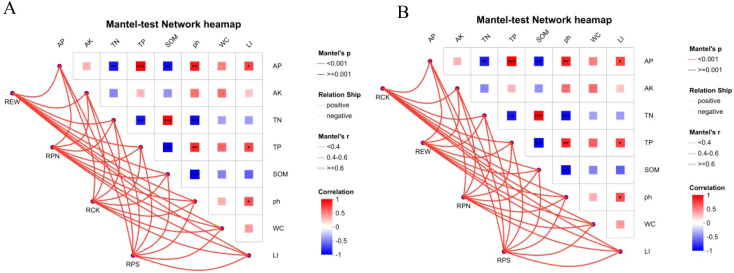
Mantel’s test analysis of soil physicochemical properties and microbial communities. **(A)** Mantel’s test results for bacterial communities. **(B)** Mantel’s test results for fungal communities.

The correlations between the top five microorganisms in terms of abundance and soil environmental factors, including pH, organic matter, fast-acting phosphorus (FAP), fast-acting potassium (FAPK), total nitrogen (TN), and total phosphorus (TP), were analyzed using Mantel’s test network heatmap (**Figures 7A, B**). The results showed that bacteria and fungi exhibited significant differentiation among different woodland soil treatments and differences in environmental factors affecting these variations. Redundancy Analysis (RDA) analyses further revealed that the top two constraint axes (RDA1 and RDA2) for bacteria explained 24.56% and 9.54% of the variance, respectively. Among them, Acidobacteriales and total phosphorus had the highest correlation, Subgroup-2 had the strongest correlation with fast-acting potassium, and *Bradyrhizobium* and organic matter were significantly correlated. For fungi, RDA1 and RDA2 explained 19.65% and 10.29%, respectively. *Saitozyma* and *Solicoccozyma* showed the highest correlation for total phosphorus, while *Sebacina* had the highest correlation with total nitrogen, as well as *Penicillium* and quicklime phosphorus.

### 3.3 Mechanisms of root secretion effects on rhizosphere microbial communities in understory medicinal plants

#### 3.3.1 Integrated consistency analysis of root secretion effects on rhizosphere microbial communities

The following figure demonstrates the study of the relationship between the microbiome and metabolome datasets via Procrustes analysis and Orthogonal Partial Least Squares (O2PLS) score plots covering comparisons of different experimental groups (RCK, RPN, RPS, and REW).

**Figure 8:**
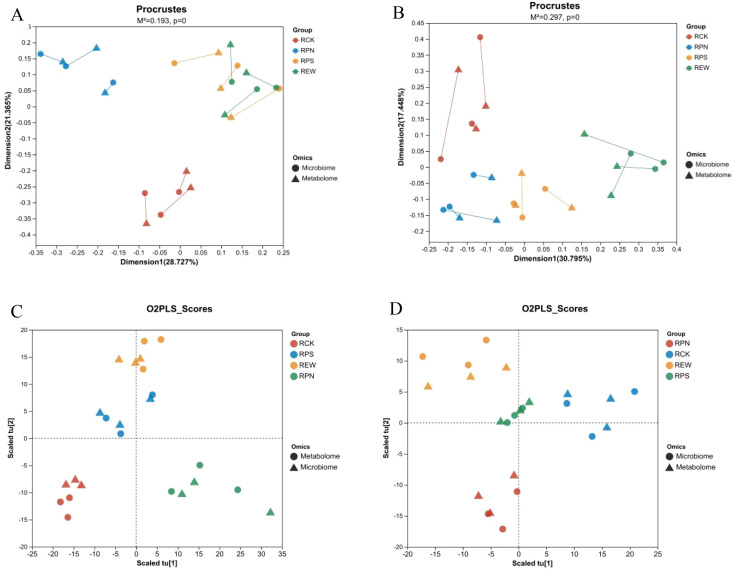
Procrustes and O2PLS score analysis of root secretion effects on rhizosphere microbial communities. **(A)** Procrustes analysis results for bacterial communities. **(B)** Procrustes analysis results for fungal communities. **(C)** O2PLS score analysis results for bacterial communities. **(D)** O2PLS score analysis results for fungal communities.

Procrustes analysis plot (M^2^ = 0.193, p = 0) showed the ordination space integrating the microbiome and metabolome datasets (**Figure 8A**). Each point represents a sample, and the connecting line highlights paired data from the two histological dimensions. The X-axis (dimension 1) explains 28.27% of the variance, and the Y-axis (dimension 2) explains 25.45% of the variance, suggesting that there is some degree of agreement between the two datasets. Procrustes analysis plot (M^2^ = 0.297, p = 0) demonstrated another set of data with dimension 1 and dimension 2 explaining 30.75% and 17.44% of the variance, respectively (**Figure 8B**). The pattern of grouping between samples showed some group differences.

The O2PLS score plot compared the metabolome and microbiome data for the RCK, RPN, RPS, and REW groups (**Figure 8C**). The plot showed cross-validated scaling latent variables (tu1 and t2), with a clear pattern of clustering between sample groups and histology data. The O2PLS score plot further emphasized the trend of separation between the two histological strata while maintaining the characteristics of the group-specific distribution (**Figure 8D**).

The results show that the microbiome and metabolism groups exhibited a strong correlation with each other via Procrustes analysis, which is further supported by the low M^2^ values and significant p-values. The O2PLS score plots validated this relationship, clearly demonstrating the clustering patterns and synergies between the groups. These results emphasize the integrated nature of microbial and metabolic responses and suggest their potential biological significance.

#### 3.3.2 Association correlation analysis of root secretion on soil inter-root microbial communities

**Figure 9:**
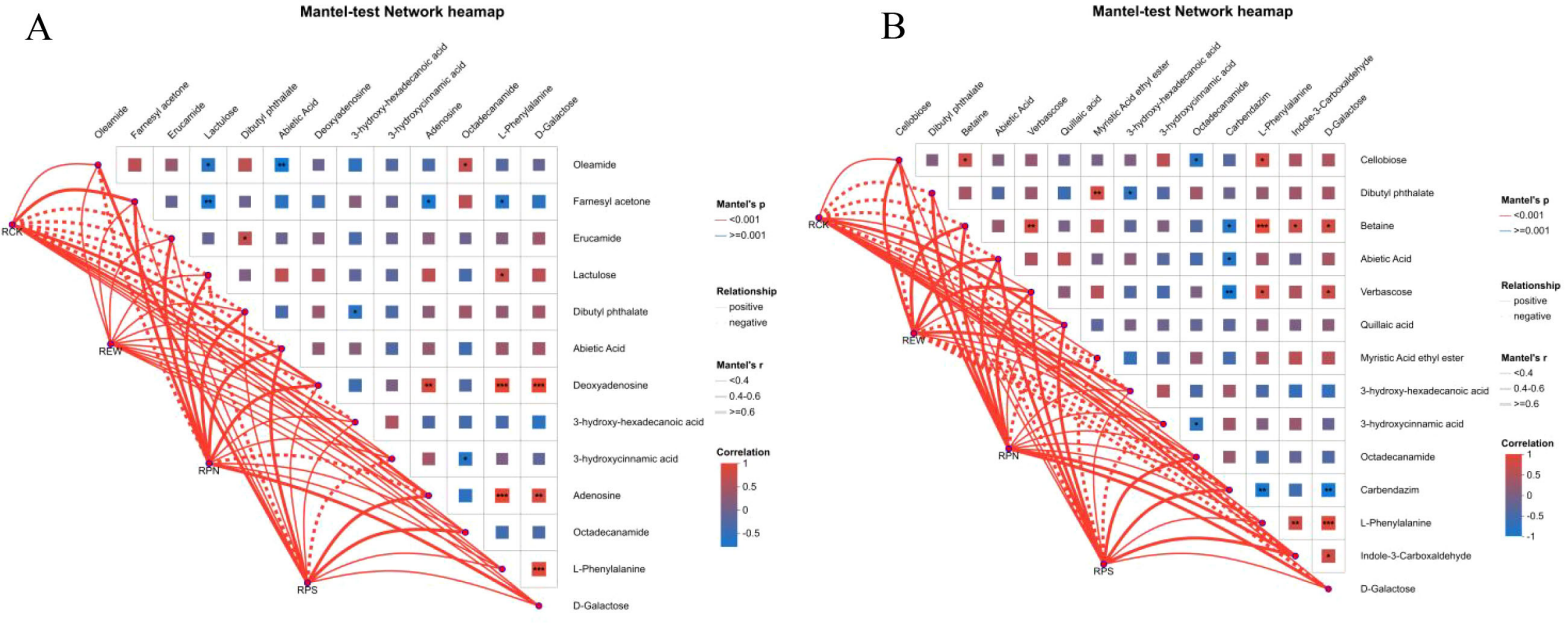
Mantel’s test analysis of root secretion effects on rhizosphere microbial communities. **(A)** Mantel’s test of root secretion effects on rhizosphere microbial communities. **(B)** Mantel’s test results for fungal communities.

The regulatory effect of root exudates on rhizosphere microbial communities was analyzed using Mantel’s test of network heatmaps. The bacterial community (**Figure 9A**) showed that the RPN group was strongly positively correlated with multiple metabolites (such as oleamide, farnesyl acetone, and erucamide) (r > 0.6, p < 0.001), indicating that this treatment significantly promoted the association between the bacterial communities and beneficial metabolites. The RPS group showed a negative correlation with lactulose, deoxyadenosine, etc. (r < −0.5, p < 0.01), indicating its inhibition of specific bacterial metabolite interactions. There was a moderate correlation (r ≈ 0.4–0.6) between the RCK control group and the REW group in metabolites such as acetic acid and l-phenylalanine, but the significance was low (p ≥ 0.001).

In the fungal community (**Figure 9B**), the RPN group showed a significant positive correlation with cellulose, dibutyl phthalate, etc. (r > 0.6, p < 0.001), while the RPS group showed a negative correlation with verbascose, quinic acid, etc. (r < −0.5, p < 0.01). Network topology analysis showed that RPN treatment enhanced community stability by strengthening microbial metabolite associations, while RPS treatment changed community structure through negative regulation, providing key evidence for elucidating the rhizosphere interaction mechanism mediated by root exudates.

**Figure 10:**
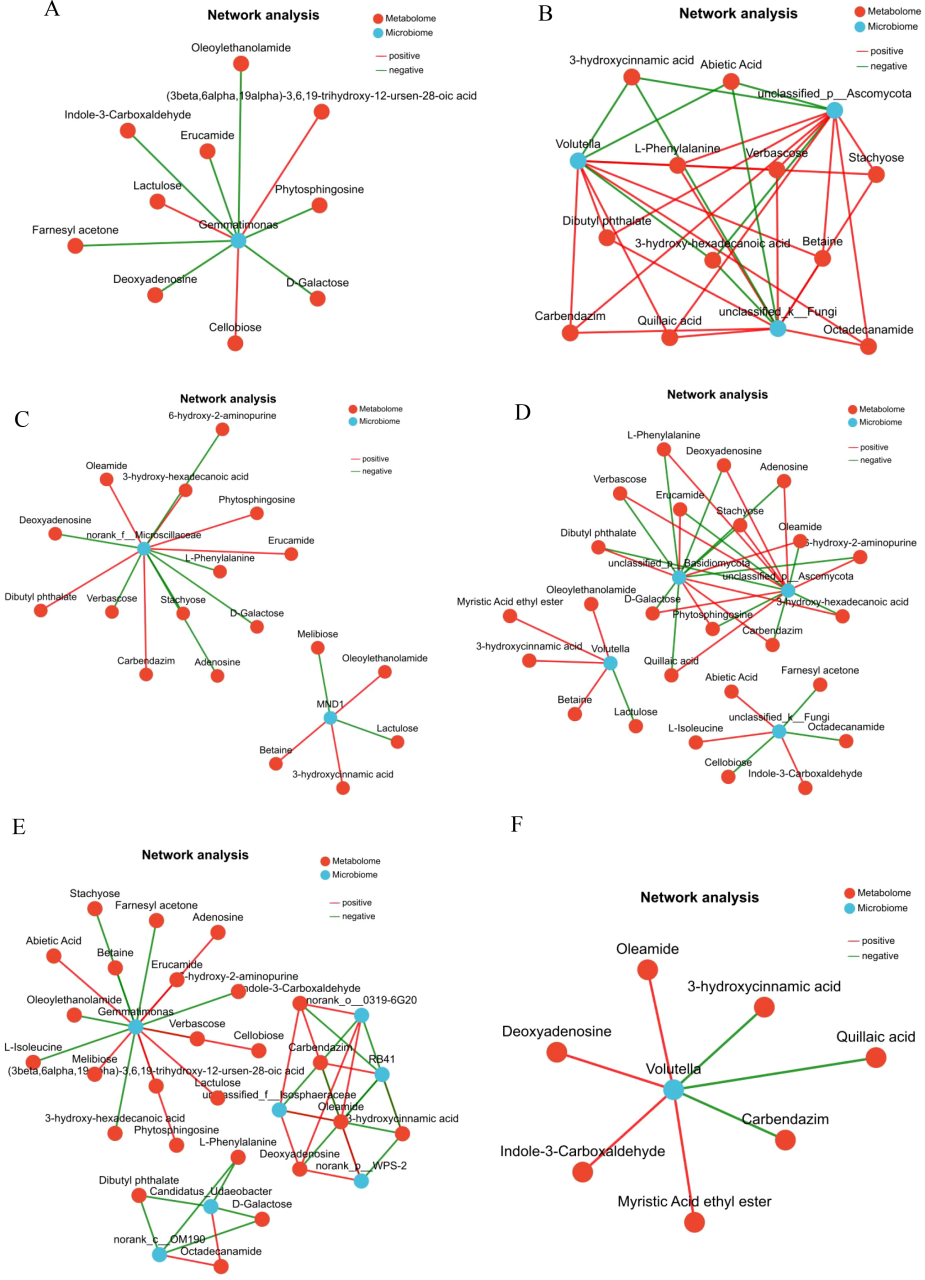
|Analysis of the correlation network diagram between root exudates and soil rhizosphere microbial community. **(A)** Correlation network diagram of RPN bacterial community. **(B)** Correlation network diagram of RPN fine fungal community. **(C)** Correlation network diagram of RPS bacterial community. **(D)** Correlation network diagram of RPS fine fungal community. **(E)** Correlation network diagram of REW bacterial community. **(F)** Correlation network diagram of REW fine fungal community.

The network interaction between rhizosphere microbial communities and metabolites under RPN treatment was analyzed. The bacterial community (**Figure 10A**) showed that *Gemmatimonas*, as the core bacterial genus, was significantly associated with multiple metabolites: oleoylethanolamide and (3beta,6alpha,19alpha)-3,6,19-trihydroxy-12-ursen-28-oic acid. Lipid compounds showed a strong positive correlation (r > 0.6, p < 0.001), while sugars such as lactulose and deoxyadenosine showed a negative correlation (r < −0.5, p < 0.01), suggesting that *Gemmatimonas* may affect plant physiology by regulating lipid metabolism. In the fungal community (**Figure 10B**), Ascomycota was positively correlated with phenolic compounds such as 3-hydroxycinnamic acid and abietic acid (r > 0.6, p < 0.001), while negatively correlated with oligosaccharides such as verbascose and stachyose (r < −0.5, p < 0.01). The *Volutella* genus formed a complex interaction network with secondary metabolites such as dibutyl phthalate and quillaic acid. Network topology analysis showed that RPN treatment promoted the accumulation of beneficial metabolites (such as phenols and lipids) by reshaping microbial metabolite associations, while inhibiting redundant carbohydrate metabolism, providing key evidence for deciphering the microbial mechanisms underlying the formation of medicinal plant quality.

The network interaction between rhizosphere microbial communities and metabolites under RPS treatment was analyzed. In the bacterial community (**Figure 10C**), *norank_f:Microscillaceae* was the core bacterial group, strongly positively correlated with lipid compounds such as oleamide and 3-hydroxy-hexadecanoic acid (r > 0.6, p < 0.001), while negatively correlated with oligosaccharides such as verbascose and stachyose (r < −0.5, p < 0.01). The *MND1* genus was negatively correlated with lactulose, betaine, etc. (r < −0.5, p < 0.01). The fungal community (**Figure 10D**) showed that Basidiomycota was positively correlated with phenolic compounds such as 3-hydroxycinnamic acid and abietic acid (r > 0.6, p < 0.001), while negatively correlated with oligosaccharides such as verbascose and stachyose (r < −0.5, p < 0.01). *Volutella* formed a complex interaction network with secondary metabolites such as dibutyl phthalate and quillaic acid. Network topology analysis showed that RPS treatment optimized the allocation strategy of rhizosphere metabolites by regulating the microbial functional niche, providing functional evidence for elucidating the microbial-driven mechanism of quality formation in medicinal plants.

The network interaction between rhizosphere microbial communities and metabolites under REW treatment was analyzed. In the bacterial community (**Figure 10E**), *Gemmatimonas*, as the core bacterial genus, showed a strong positive correlation with oligosaccharides and terpenoids such as stachyose and farnesyl acetone (r > 0.6, p < 0.001), while negative correlation with oleoylethanolamide and (3beta,6alpha,19alpha)-3,6,19-trihydroxy-12-ursen-28-oic acid. The lipid compounds showed a negative correlation (r < −0.5, p < 0.01); *Candidatus Udaeobacter* showed a negative correlation with d-galactose, Octadecanamide, and others (r < −0.5, p < 0.01). The fungal community (**Figure 10F**) showed that the *Volutella* genus was positively correlated with phenolic compounds such as oleamide and 3-hydroxycinnamic acid (r > 0.6, p < 0.001), while negatively correlated with quillaic acid, carbendazim, etc. (r < −0.5, p < 0.01). Network topology analysis showed that REW treatment promoted the accumulation of oligosaccharides and terpenoid metabolites by reshaping the microbial metabolite association, while inhibiting lipid compound synthesis, forming functional differentiation with RPN treatment, providing key evidence for elucidating the microbial mechanisms underlying the quality formation of medicinal plants under different treatments.

## 4 Discussions

Microbial community characterization is an important factor influencing soil quality and plant diseases, and there are significant differences in the composition of microbial communities in different habitats. The results of this study showed that there were significant differences in the structure and abundance of bacterial and fungal communities in natural control and plantation woodlands, and the diversity of the bacterial communities was higher than that of natural woodlands in the forested *P. notoginseng* plantation woodland, *P. sibiricum* plantation woodland, and forested *E. wasabia* plantation woodland (Zhang et al., 2023; Li et al., 2024). The root secretions, apoplastic materials, and inter-root sediments of the understory herbs provided abundant carbon sources and energy for the inter-root microorganisms, among which the root secretions of the understory herbs *P. notoginseng*, *P. sibiricum*, and *E. wasabia* were rich in components, which mainly included lipids, organic acids, phenylpropanoids, and polyketides (Wang et al., 2022; Chen et al., 2025). The root secretions of *P. notoginseng* and *E. wasabia* were also rich in components. These components play important roles in plant growth and development, disease resistance, and soil microbial activity, especially lipids and organic acids dominate in their metabolic processes. This may increase the diversity of inter-root microorganisms and support the survival of more species of microorganisms (Liu et al., 2023). Meanwhile, components such as organic acids and sugars in the root secretions of herbal medicines can significantly affect the growth and metabolism of soil microorganisms, which promotes the healthy development of soil ecosystems and stimulates the metabolic activity of microorganisms, which in turn affects the transformation and cycling of soil nutrients (Zhao et al., 2024). *P. notoginseng*, *P. sibiricum*, and *E. wasabia* are perennial no-till continuous crops, and prolonged high-density monoculture may lead to the accumulation of pathogenic bacteria and reduction of soil microbial diversity, which may lead to serious soil-borne root rot problems (Sun et al., 2021; Zhou et al., 2025).

In this study, we showed that pathogenic bacteria such as *Bradyrhizobium*, *Saitozyma*, and *Sebacina*, the presence of which may affect the resistance of herbal medicines to soil-borne diseases, are closely related to the changes in the inter-root microbial community (Gao et al., 2022; Wu et al., 2023a). These pathogenic bacteria may lead to a decrease in the resistance of herbal plants to soil-borne diseases, making the plants more susceptible to disease attack and affecting their growth and yield (Huang et al., 2024). Meanwhile, the presence of pathogenic bacteria may lead to changes in the structure of the inter-root microbial community and inhibit the growth of beneficial microorganisms, thus affecting the ecological balance of the soil (Deng et al., 2020). In addition, pathogenic bacteria may infect the plant root system, leading to diseases such as root rot, which affects the plant’s ability to absorb water and nutrients, thereby affecting overall growth (Xu et al., 2021). Changes in the inter-root microbial community may also affect the transformation and cycling of nutrients in the soil, which in turn affects soil fertility and plant nutritional status (Yan et al., 2022). Plant growth and development may be inhibited due to pathogenic bacteria, leading to problems such as slow growth, yellowing of leaves, and reduced flowering and fruiting (Tang et al., 2023). Finally, changes in the inter-root microbial community may affect ecosystem services such as water retention, carbon sequestration, and nitrogen fixation in the soil, which in turn may negatively affect the health of the entire ecosystem (Ma et al., 2024). In addition, the cultivation of herbal medicines may change the physicochemical properties of the soil, such as pH, nutrient content, and porosity, which in turn affects the microbial survival environment and enzyme activities (Zhang et al., 2021). It may be possible to reduce the abundance of dominant flora to prevent and control root rot diseases of Chinese medicinal herbs, and at the same time, in terms of planting management, optimizing the planting pattern of understory Chinese medicinal herbs (e.g., monoculture planting, intercropping, and intercropping) may help to increase the diversity and activity of inter-root microorganisms, thus promoting the maintenance and improvement of soil fertility (He et al., 2025). Studies have shown that an appropriate increase in soil nutrients and water can help increase the content of active components in root secretions and enhance the ecological adaptability of plants (Lin et al., 2020).

The interaction between underground rhizosphere microbial communities and root exudates in *P. notoginseng*, *P. sibiricum*, and *E. wasabia* plantations was analyzed using Mantel’s network heatmap system, revealing the differential regulatory mechanisms of microbial metabolite association patterns under different treatments (Qin et al., 2024a). In the *P. notoginseng* plantation forest, *Gemmatimonas* (bacteria) showed a strong positive correlation with lipid compounds (oleoylethanolamide, etc.) (r > 0.6, p < 0.001), while Ascomycota (fungi) showed a significant correlation with phenolic compounds (3-hydroxycinnamic acid, etc.), indicating that this treatment improved plant quality by promoting the accumulation of lipids and phenolic metabolites (Liu et al., 2021). In the *P. sibiricum* plantation forest, *norank_f:Microscillaceae* (bacteria) was positively correlated with lipid compounds and negatively correlated with oligosaccharides, while Basidiomycota (fungi) was positively correlated with phenolic compounds, indicating its optimization effect on microbial functional niche (Wang et al., 2023). The planting of *E. wasabia* forest presents the opposite pattern, with *Gemmatimonas* (bacteria) positively correlated with oligosaccharides and terpenoids and negatively correlated with lipids, while *Volutella* (fungi) positively correlated with phenolic compounds and negatively correlated with quillaic acid, forming functional differentiation with *P. notoginseng* forest treatment (Chen et al., 2025). The three treatments reshaped the microbial metabolite association network to regulate the accumulation of metabolites such as lipids, phenols, and oligosaccharides, providing key evidence for understanding the microbial-driven mechanisms underlying the quality formation of medicinal plants (Zhang et al., 2021).

In summary, the effects of root secretions of understory herbs on the characteristics of inter-root microbial communities are of great ecological significance. Future studies should focus on the precise identification of root secretion components, the dynamic monitoring of microbial communities, and the comprehensive consideration of environmental factors, with a view to providing a scientific basis for the sustainable cultivation of Chinese herbal medicines (Li et al., 2024). These studies not only provide an important basis for optimizing the planting pattern of Chinese herbs and improving the quality of herbs but also lay a foundation for promoting the sustainable development of forest economy (Huang et al., 2023).

## 5 Conclusions

1. The chemical characteristics of root exudates exhibited significant species specificity: *P. notoginseng*, *P. sibiricum*, and *E. wasabia* secreted 329, 250, and 193 compounds, respectively. Among them, *P. notoginseng* and *E. wasabia* share 165 different secretions. LC-MS analysis showed that *P. notoginseng* is mainly composed of lipids and lipid-like molecules (238 species), while *P. sibiricum* (139 species) and *E. wasabia* (162 species) have a high proportion of lipid molecules but different composition types, reflecting the differences in plant ecological functions and pharmacological effects.
2. The composition and diversity of microbial communities are regulated by root exudates: the abundance and diversity indices of Proteobacteria, Acidobacteria (bacterial dominant phylum), and Basidiomycota (fungal dominant phylum) are affected by exudates. Mantel’s test confirmed that the main components of exudates are significantly positively correlated with microbial communities (r > 0.6, p < 0.001), promoting microbial growth and functional performance.
3. Enrichment of metabolic pathways showed plant adaptation strategies: *P. notoginseng* forest enriched 22 metabolic pathways (15 significant pathways); *P. sibiricum* and *E. wasabia* enriched 20 and 15 pathways, respectively (including multiple significant pathways). KEGG analysis revealed that different plants adapt to the environment through differentiated metabolic pathways, such as *P. notoginseng* promoting lipid metabolism and *P. sibiricum* optimizing functional niche.
4. The microbial metabolite interaction network has processing specificity: *Gemmatimonas* (bacteria) in *P. notoginseng* forest was positively correlated with lipids, and Ascomycota (fungi) was positively correlated with phenols; *norank_f:Microscillaceae* (bacteria) was positively correlated with lipids, while Basidiomycota (fungi) was positively correlated with phenols in *P. sibiricum* forest land; *Gemmatimonas* (bacteria) in *E. wasabia* forest was positively correlated with oligosaccharides/terpenes, and *Volutella* (fungi) was positively correlated with phenols. The three treatments regulated the accumulation of different metabolites by reshaping the associated network.
5. Ecological significance and sustainable agricultural applications: Root exudates affect microbial communities through nutrient supply and chemical signals, promote beneficial microbial growth, enhance metabolic activity, provide a basis for analyzing the mechanism of medicinal plant quality formation, emphasize the importance of plant–microbe interaction in ecosystem management, and lay the foundation for optimizing soil microbial health and sustainable agriculture.

## Data Availability

The datasets presented in this study can be found in online repositories. The names of the repository/repositories and accession number(s) can be found in the article/**Supplementary Material**.
