# Supplementary figures and images for: Influence of root secretions of understory Chinese herbal medicines on the characterization of inter-root microbial communities
Source: Front Plant Sci. 2026 Jan 20;16:1697347. doi: 10.3389/fpls.2025.1697347 (PMC12864437; doi:10.3389/fpls.2025.1697347)

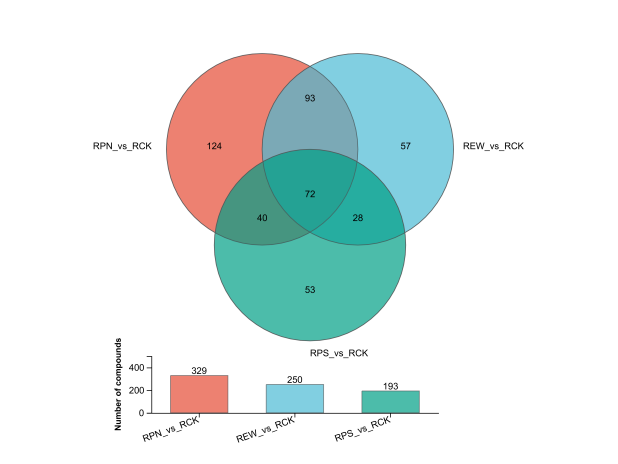

Supplement: Supplementary file 1 [file DataSheet1.zip › image/1.png]

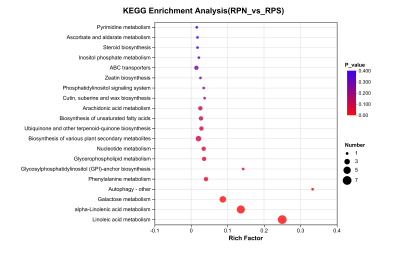

Supplement: Supplementary file 1 [file DataSheet1.zip › image/10.png]

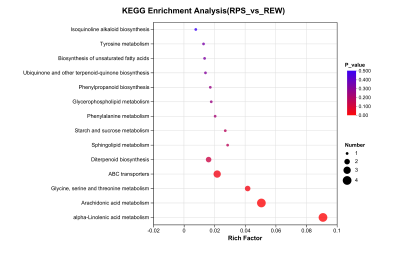

Supplement: Supplementary file 1 [file DataSheet1.zip › image/11.png]

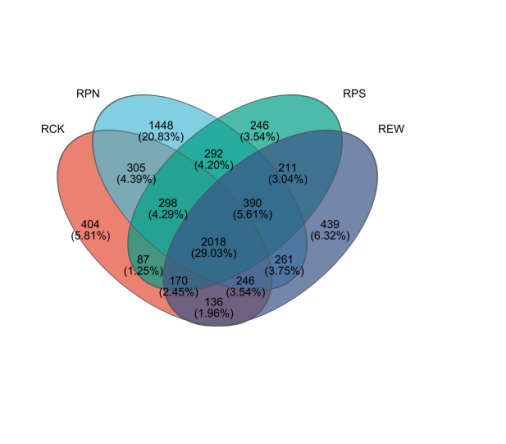

Supplement: Supplementary file 1 [file DataSheet1.zip › image/12.png]

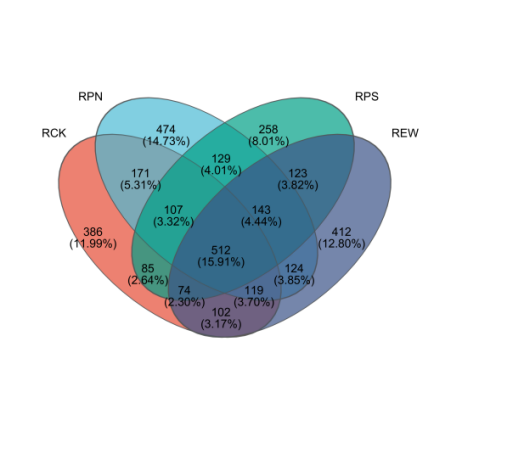

Supplement: Supplementary file 1 [file DataSheet1.zip › image/13.png]

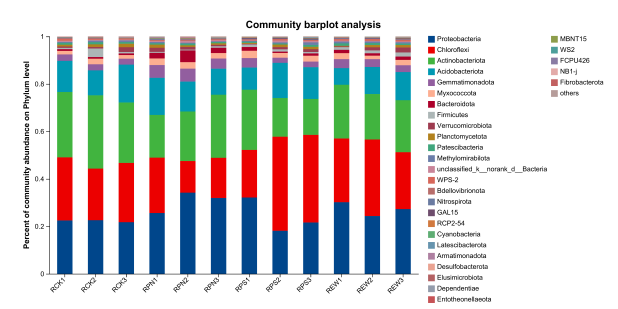

Supplement: Supplementary file 1 [file DataSheet1.zip › image/14.png]

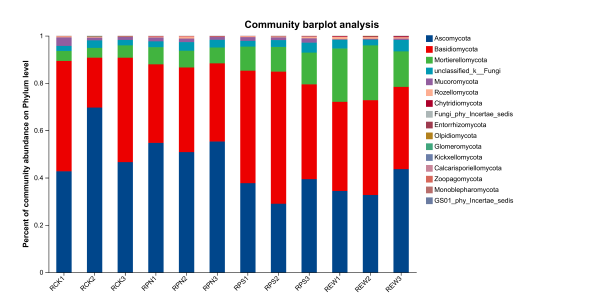

Supplement: Supplementary file 1 [file DataSheet1.zip › image/15.png]

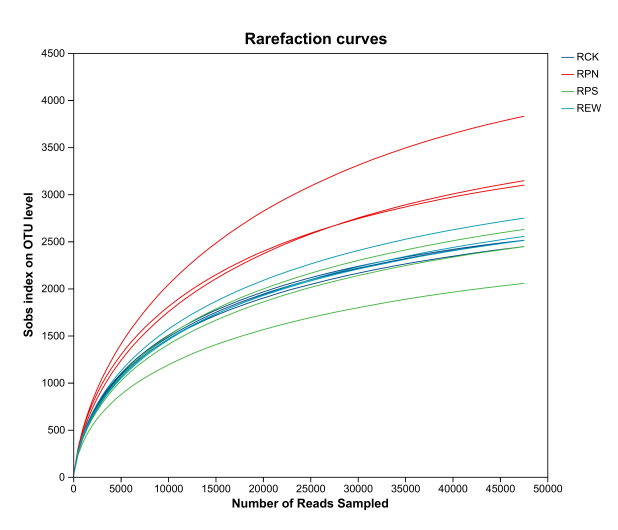

Supplement: Supplementary file 1 [file DataSheet1.zip › image/16.png]

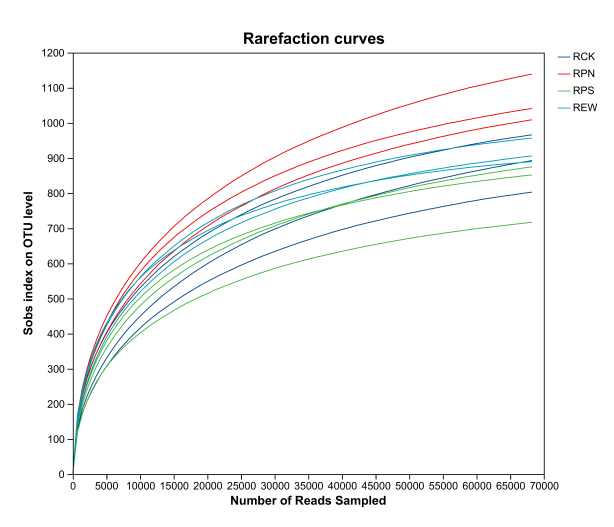

Supplement: Supplementary file 1 [file DataSheet1.zip › image/17.png]

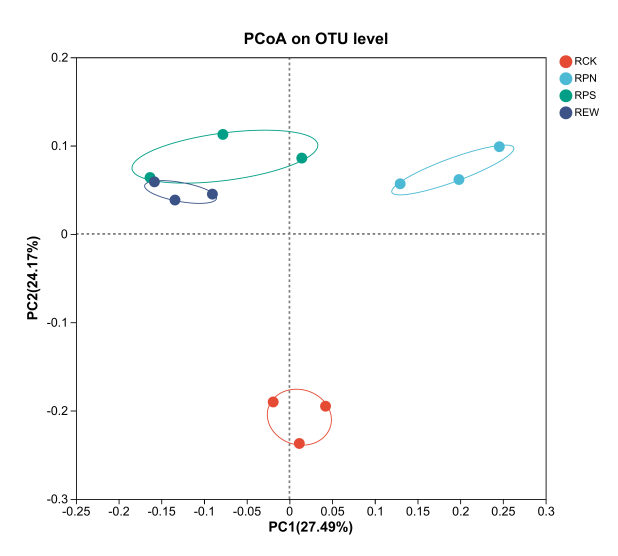

Supplement: Supplementary file 1 [file DataSheet1.zip › image/18.png]

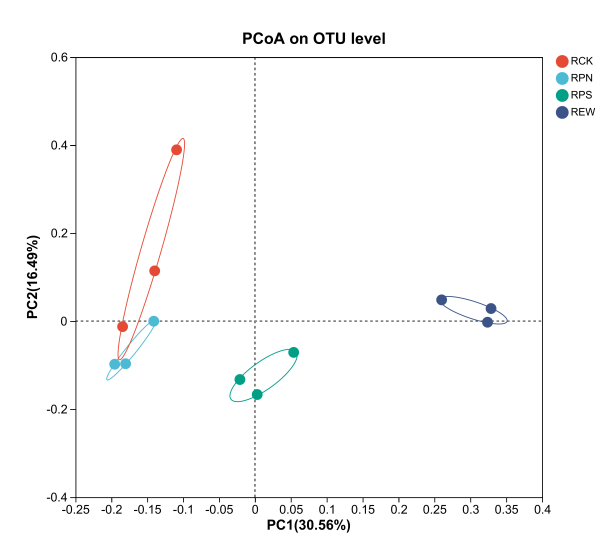

Supplement: Supplementary file 1 [file DataSheet1.zip › image/19.png]

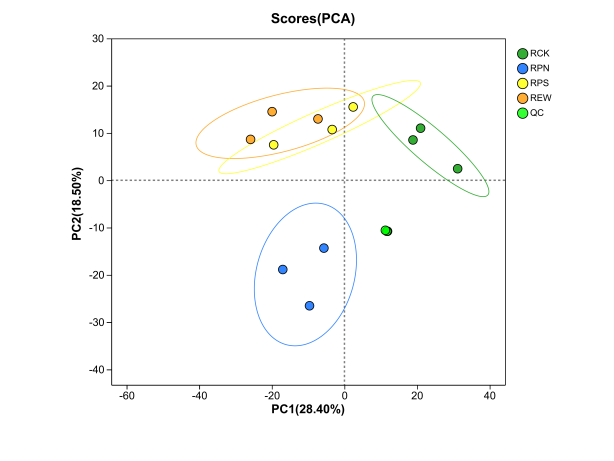

Supplement: Supplementary file 1 [file DataSheet1.zip › image/2.png]

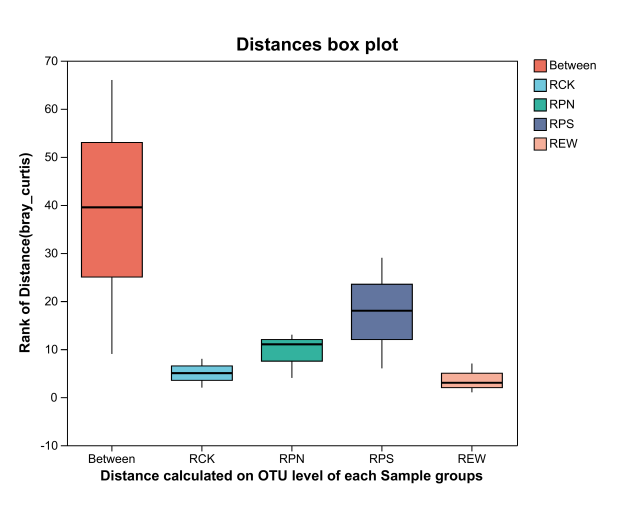

Supplement: Supplementary file 1 [file DataSheet1.zip › image/20.png]

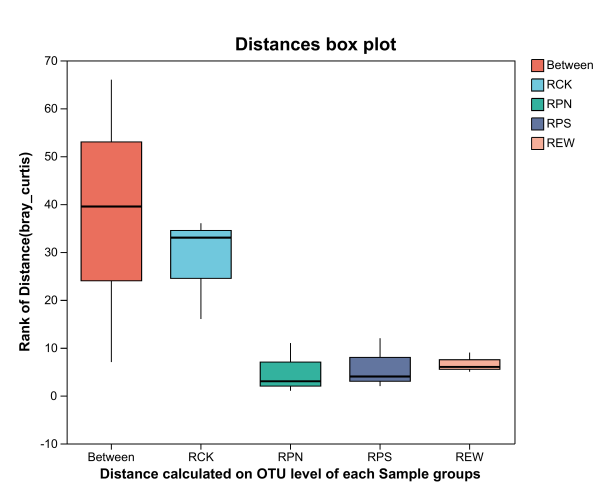

Supplement: Supplementary file 1 [file DataSheet1.zip › image/21.png]

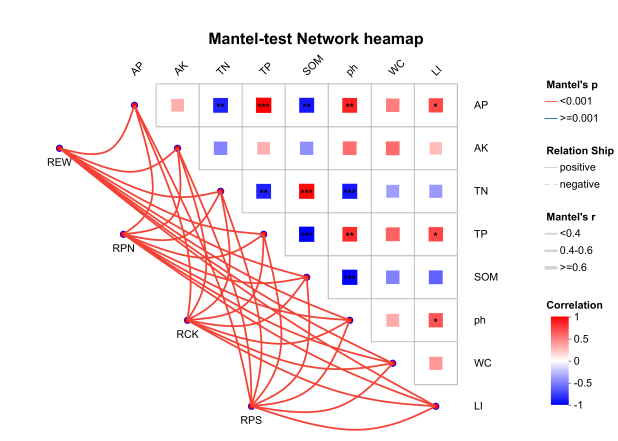

Supplement: Supplementary file 1 [file DataSheet1.zip › image/22.png]

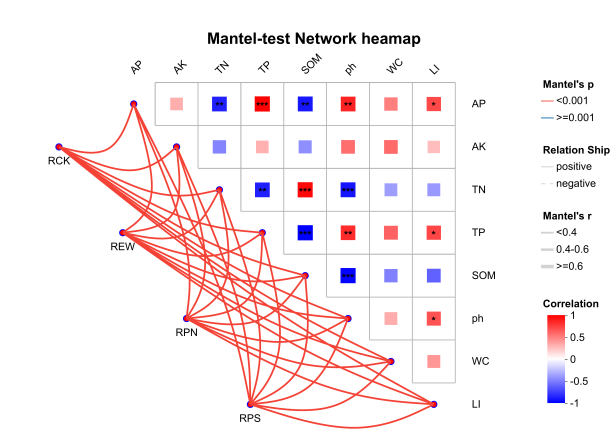

Supplement: Supplementary file 1 [file DataSheet1.zip › image/23.png]

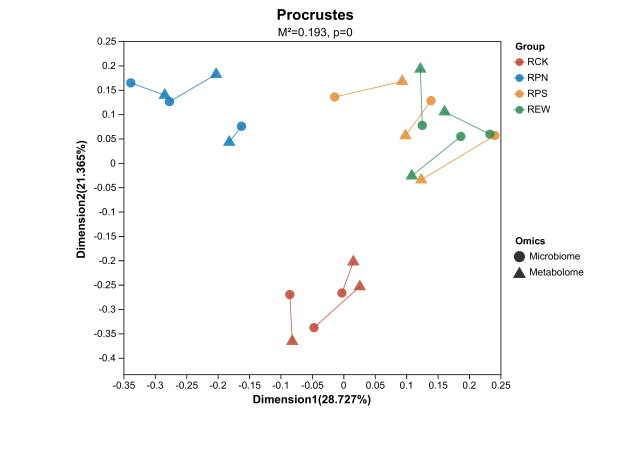

Supplement: Supplementary file 1 [file DataSheet1.zip › image/24.png]

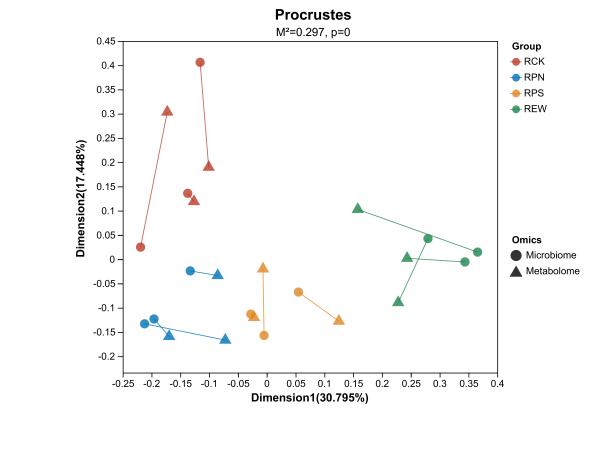

Supplement: Supplementary file 1 [file DataSheet1.zip › image/25.png]

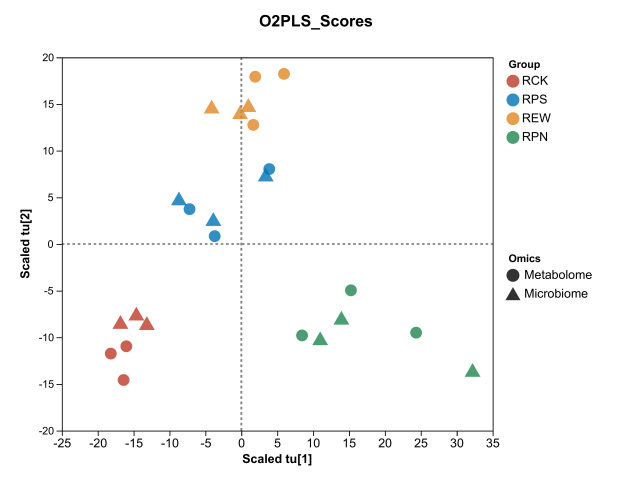

Supplement: Supplementary file 1 [file DataSheet1.zip › image/26.png]

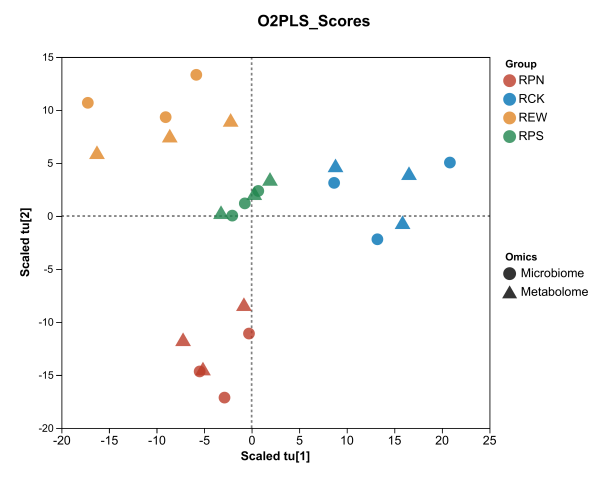

Supplement: Supplementary file 1 [file DataSheet1.zip › image/27.png]

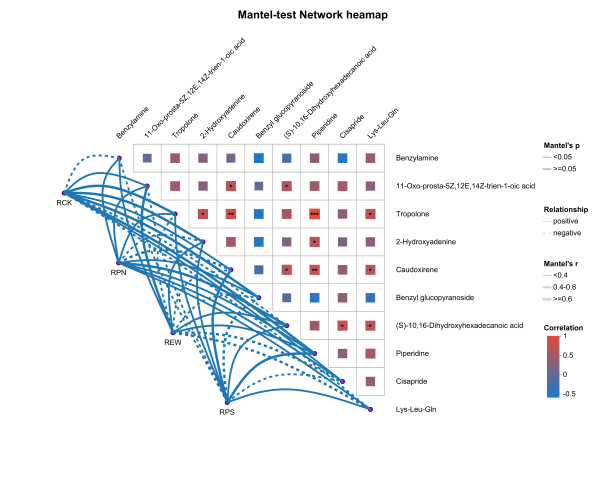

Supplement: Supplementary file 1 [file DataSheet1.zip › image/28.png]

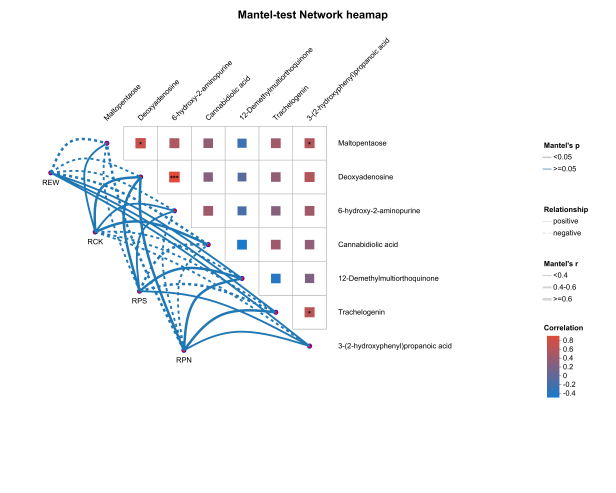

Supplement: Supplementary file 1 [file DataSheet1.zip › image/29.png]

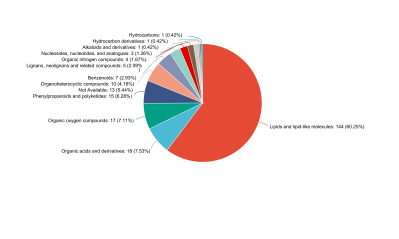

Supplement: Supplementary file 1 [file DataSheet1.zip › image/3.png]

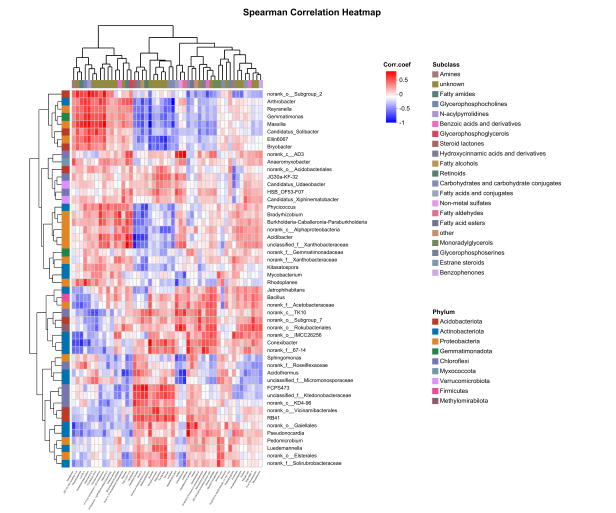

Supplement: Supplementary file 1 [file DataSheet1.zip › image/30.png]

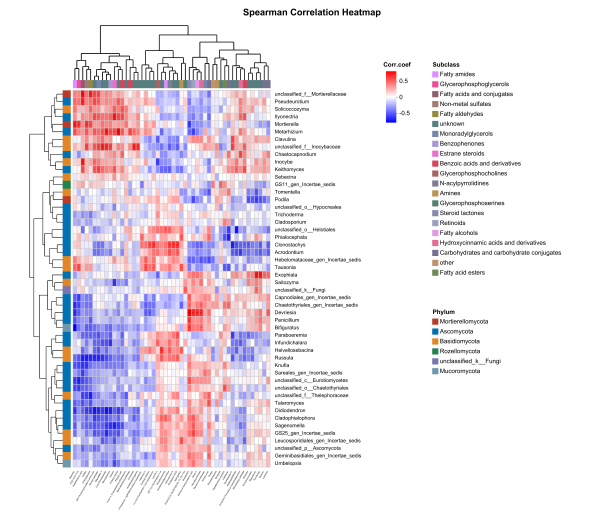

Supplement: Supplementary file 1 [file DataSheet1.zip › image/31.png]

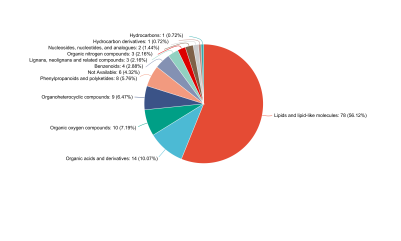

Supplement: Supplementary file 1 [file DataSheet1.zip › image/4.png]

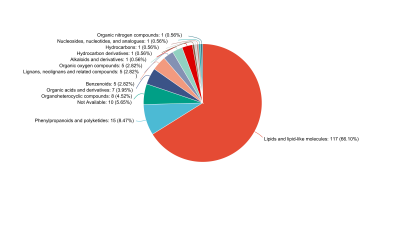

Supplement: Supplementary file 1 [file DataSheet1.zip › image/5.png]

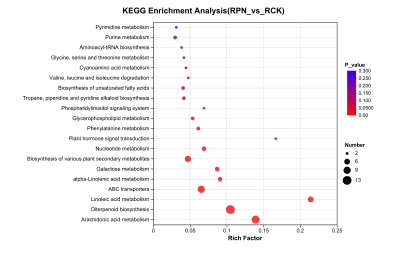

Supplement: Supplementary file 1 [file DataSheet1.zip › image/6.png]

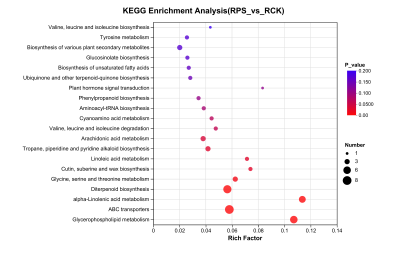

Supplement: Supplementary file 1 [file DataSheet1.zip › image/7.png]

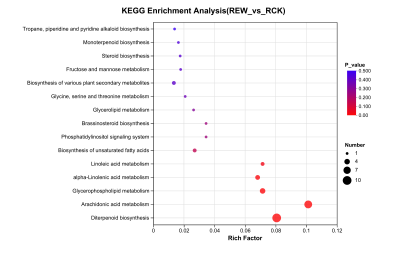

Supplement: Supplementary file 1 [file DataSheet1.zip › image/8.png]

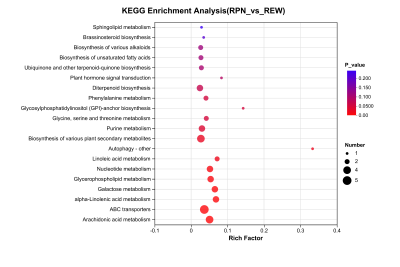

Supplement: Supplementary file 1 [file DataSheet1.zip › image/9.png]
