# Supplementary material for: Influence of root secretions of understory Chinese herbal medicines on the characterization of inter-root microbial communities
Source: Front Plant Sci. 2026 Jan 20;16:1697347. doi: 10.3389/fpls.2025.1697347 (PMC12864437; doi:10.3389/fpls.2025.1697347)
Supplement: Supplementary file 2 [file Table1.docx]

https://www.scidb.cn/anonymous/WnJpdU1y
